# "Stop, You’re Killing us!" An Alternative Take on Populism and Public Health: Comment on "The Rise of Post-truth Populism in Pluralist Liberal Democracies: Challenges for Health Policy"

**DOI:** 10.15171/ijhpm.2017.50

**Published:** 2017-04-26

**Authors:** Ted Schrecker

**Affiliations:** School of Medicine, Pharmacy and Health, Durham University, Stockton-on-Tees, UK.

**Keywords:** Populism, Post-democracy, Science, Standards of Proof

## Abstract

Ewen Speed and Russell Mannion correctly identify several contours of the challenges for health policy in what it is useful to think of as a post-democratic era. I argue that the problem for public health is not populism per se, but rather the distinctive populism of the right coupled with the failure of the left to develop compelling counternarratives. Further, defences of ‘science’ must be tempered by recognition of the unavoidably political dimensions of the (mis)use of scientific findings in public policy.

Ewen Speed and Russell Mannion^1^ correctly and eloquently identify several contours of the challenges for health policy in what it is useful to think of as a post-democratic era. Citing the crucial work of Inglehart and Norris,^2^ they perform the indispensable service of foregrounding for health researchers and practitioners a process that blind-sided many of us (myself included), even though Zakaria warned two decades ago about the rise of ‘illiberal democracy’^3^; Freedom House identified the year 2006 as a turning point^4^; and Diamond – one of the founding editors of *Journal of Democracy*, which these days is well worth reading – explicated multiple manifestations of the trend in 2015.^5^

Populism is a complicated if not Protean category. I would argue that the problem for health policy is not populism *per se* but rather, in many political contexts, the ability of key protagonists in the transnational capitalist class^6-9^ and allied domestic elites to misdirect^ [1]^ the identification of threats to the health and well-being of populations left behind by neoliberal economic integration. Predictably in view of the financial interests of the key backers of Brexit and (more visibly) the Trump campaign, neither targeted fraudster bankers, billionaire hedge fund investors in private equity, or the acolytes of ‘shareholder value’^10,11^ whose activities especially in the United States drove the destruction of industrial jobs. Unfortunately, these protagonists were also largely ignored by the mainstream political left. In the UK context, the most conspicuous issue is the Labour Party’s catastrophic failure to challenge the Conservative ascription of the post-2008 financial crisis and recession to Labour’s economic policies, rather than to politically protected corporate crime on the other side of the Atlantic. In the United States, absent a competing and credible narrative that challenged the role of corporate capital in spreading economic insecurity and inequality^ [2],12^ the remarkable turn to Trump among the left-behind – those whose health status is most clearly endangered by ‘neoliberal epidemics,’^13^ as *The Economist* pointed out after the election^14^ – is lamentable but hardly surprising.

We can and should envision an alternative populism, organised around a rubric like: ‘Stop, you’re killing us!’ This is not original; it is adapted from the slogan of the ‘Trainites’: cells of environmental activists inspired, although not organised, by an environmental scientist named Austin Train in John Brunner’s dystopian 1972 novel *The Sheep Look Up*.^15^ Brunner anticipated a near-future of ecological collapse, against the background of a bellicose and domestically callous US government led by a buffoonish president (‘Prexy’) who bears an uncanny resemblance to one Donald Trump. Sometimes, the fictional past is factual prologue. This form of populism has emerged in some media coverage (Figure) of a recent finding that as the number of frail older people receiving social care from local governments in the United Kingdom fell by 300 000 post-2010,^16^ the number of deaths in England and Wales increased by 39 074 in the year ending July, 2015 over the preceding year, with 32 208 of those deaths occurring among those aged 80 and older.^17^ The straightforward insight conveyed by the headline in the figure remains, for the moment, outside the political mainstream – a point of special concern in the election campaign that at this writing is under way in the United Kingdom. Researchers and practitioners who care about health inequalities and their economic and political substrates should welcome this form of populism; ally ourselves with those who can communicate the message to a broader audience; and do the best we can to ensure the intellectual integrity both of the relevant findings and of the way they are used in public policy and political advocacy.

**Figure:**
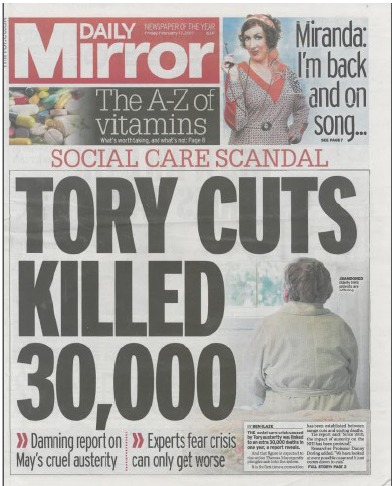
An Alternative Form of Populism.

That said, the politics of science and evidence in a ‘post-truth world’ are unavoidably complicated. The tactic of ‘manufacturing uncertainty’ has a long and ignoble history, perfected by the tobacco industry and also deployed in defence of various environmental, workplace and consumer product contaminants,^18-20^ conspicuously in recent efforts to resist European Union (EU) regulation of endocrine-disrupting chemicals.^21^ When Trump administration officials deny causal connections between human activity and climate change, in the face of findings from the largest-ever multinational scientific collaboration,^22^ it is tempting simply to defend ‘science.’ Efforts to undermine the integrity of scientific investigation or to restrict scientific researchers’ public disclosure of their findings, as under the Canadian government of Conservative Stephen Harper, must always be resisted. At the same time, we must recognise that the production of scientific knowledge is a social process, and that there is a political dimension to how public policy deals with scientific uncertainty or indeterminacy.

More explicit reflection on this latter point is especially valuable. For example, the demand for epidemiological evidence of health effects from environmental risks can be cloaked in the rhetoric of good science, and industrial firms and their front organisations have often used this tactic – a variant of manufacturing uncertainty – to resist regulation. A more accurate description, as environmental economist Talbot Page pointed out long ago, is of an approach that ‘requires positive evidence of “dead bodies” before acting.’^23^ The approach neglects such issues as the difficulties associated with demonstrating statistical significance at the conventional (but entirely arbitrary) 95% confidence level, because of statistical power limitations driven by sample sizes and effect sizes. Choices about what represents a ‘conservative’ approach to scientific evidence in such situations, as for example in the broader context of the effects of austerity on health, are inescapably value-driven and political.^24^

## Ethical issues

Not applicable.

## Competing interests

Author declares that he has no competing interests.

## Author’s contribution

TS is the single author of the paper.

## Endnotes


[1] Misdirection is a term drawn from stage magic, in which the performer directs the audience’s attention elsewhere whilst the necessary sleight-of-hand is carried out. Its relevance in the current context is obvious.

[2] In 1993, for example, investment banker Steven Rattner defended massive industrial job losses, and pay cuts in those that remained, as the necessary price of moving to a high-productivity economy, noting that such an economy brings ‘the promise of higher incomes for more efficient workers and fewer jobs for everyone else.’

